# Unraveling the Role of Zonulin in Allogeneic Hematopoietic Stem Cell Transplantation: A Multicenter Study

**DOI:** 10.3390/ijms27114659

**Published:** 2026-05-22

**Authors:** Alexandre Soares Ferreira Junior, Nathalia Linares Silva, Danielle Amanda Niz Alvarez, Larissa da Silva Souza, Luiza Dias Machado, Bianca Fernanda Rodrigues da Silva, Welinton Yoshio Hirai, Rozana Mesquita Ciconelli, Joao Victor Piccolo Feliciano, Iago Colturato, George Maurício Navarro Barros, Phillip Scheinberg, Gislane Lelis Vilela de Oliveira

**Affiliations:** 1 Department of Genetics, Microbiology and Immunology, Institute of Biosciences (IBB), Sao Paulo State University (UNESP), Botucatu 18618-970, Sao Paulo, Brazil; alexandre.soares@unesp.br (A.S.F.J.); nathalia.linares@unesp.br (N.L.S.); danielle.alvarez@unesp.br (D.A.N.A.); larissa.silva-souza@unesp.br (L.d.S.S.); luiza.d.machado@unesp.br (L.D.M.); bianca-fernanda.silva@unesp.br (B.F.R.d.S.); 2 Department of Epidemiology and Biostatistics, Hospital de Amor de Barretos, Barretos 14784-400, Sao Paulo, Brazil; welinton.hirai@hospitaldeamor.com.br; 3 Departamento de Pesquisa da BP—A Beneficência Portuguesa de São Paulo, São Paulo 01323-001, Sao Paulo, Brazil; rozana.ciconelli@bp.org.br (R.M.C.);; 4 Fundação Faculdade Regional de Medicina de São José do Rio Preto (FUNFARME), São José do Rio Preto 15090-000, Sao Paulo, Brazil; joao.feliciano@hospitaldebase.com.br; 5 Hospital Amaral Carvalho, Jaú 17210-070, Sao Paulo, Brazil; 6 Fundação Pio XII—Hospital de Câncer de Barretos, Barretos 14784-400, Sao Paulo, Brazil

**Keywords:** intestinal barrier function, stem cell transplantation, gastrointestinal microbiome, intestinal mucosa, graft-versus-host-disease, zonulin

## Abstract

The role of zonulin as a biomarker of intestinal permeability in the allogeneic hematopoietic stem cell transplantation (allo-HSCT) setting remains poorly understood. In this study, we aimed to evaluate serum zonulin dynamics, identify its predictors, and assess its prognostic significance in patients undergoing allo-HSCT. This multicenter, prospective cohort study was conducted across four Brazilian hospitals. Eligible participants were patients aged ≥12 years who provided at least one blood sample during the allo-HSCT course. A control group of 15 healthy adult individuals was also included. Serum zonulin levels were quantified using enzyme-linked immunosorbent assay multiple times over the allo-HSCT course. Outcomes included acute graft-versus-host disease, overall survival, and bloodstream infections. A total of 477 blood samples were collected from 140 patients. Compared with the control group, zonulin levels were persistently elevated at all evaluated time points throughout the allo-HSCT course. However, no significant differences were observed among the different time points assessed during transplantation. No clinical or transplantation-related characteristics were identified as significant predictors of elevated zonulin levels. Finally, zonulin did not demonstrate prognostic value for allo-HSCT-related outcomes. Future studies should investigate whether other intestinal permeability biomarkers have prognostic relevance in the allo-HSCT setting.

## 1. Introduction

In patients undergoing allogeneic hematopoietic stem cell transplantation (allo-HSCT), adverse clinical outcomes, including graft-versus-host disease (GvHD) and oral mucositis, have been associated with damage to the gastrointestinal barrier [1,2,3]. The gastrointestinal barrier is a complex and dynamic structure organized into three main layers: (1) the mucus layer; (2) the intestinal epithelium; and (3) the lamina propria. These layers act synergistically to control intestinal permeability through both transcellular and paracellular pathways, thereby maintaining selective permeability (regulated transport of microorganisms, metabolites, and antigens) [4,5,6]. The paracellular pathway, which involves transport through the intercellular spaces between adjacent epithelial cells, has been classically implicated in the development of several disease states [5,7]. This route is primarily regulated by tight junctions, which are multiprotein complexes. Among the factors involved in the regulation of the paracellular route, a key modulator of tight junction integrity that promotes increased paracellular permeability is zonulin [8].

Zonulin, also known as pre-haptoglobin 2, is a multifunctional protein produced by intestinal epithelial cells that acts similarly to the Zot enterotoxin produced by *Vibrio cholerae* [7]. Zonulin increases paracellular permeability by activating epidermal growth factor (EGF) receptors in enterocytes and promoting tight junction disassembly [7]. Zonulin has been increasingly implicated in a broad spectrum of immune-mediated, metabolic, and inflammatory conditions [7]. By modulating the paracellular pathway, zonulin regulates the trafficking of multiple potential antigens and influences the balance between immune tolerance and alloreactivity to non-self antigens [7]. For instance, in patients with celiac disease, accumulating evidence has identified zonulin as an important factor in disease pathogenesis [7]. In genetically susceptible individuals, zonulin expression is upregulated in response to gluten ingestion, leading to increased paracellular permeability and enhanced exposure of the immune system to luminal antigens [7]. Zonulin has also been investigated as a potential diagnostic and prognostic biomarker. In patients with cirrhosis, zonulin levels have been shown to predict the severity of hepatic decompensation, as well as the risk of acute kidney injury and hepatorenal syndrome [9]. Other diseases that have been associated with zonulin levels include type 1 diabetes, obesity, metabolic syndrome, inflammatory bowel diseases, and cancer [7,10].

Although zonulin has been extensively evaluated in other clinical settings, its significance in patients with hematologic malignancies and those undergoing HSCT (auto and allo-HSCT) remain largely unknown. Prior studies have primarily focused on pediatric allo-HSCT patients and animal models [11,12]. The evaluation of zonulin in allo-HSCT is particularly relevant, as these patients frequently experience substantial gastrointestinal barrier damage due to multiple transplantation-related factors, including conditioning regimens, the use of total body irradiation, and intestinal dysbiosis [2,13,14,15,16]. Moreover, allo-HSCT continues to be associated with considerable morbidity and mortality. Finally, zonulin could be readily translated into clinical practice, as it represents an accessible and easily measurable surrogate marker of intestinal permeability [7,17]. Thus, the assessment of zonulin may contribute to risk stratification, outcome prediction, and the identification of potential therapeutic targets. In this multicenter Brazilian study, we aimed to evaluate the dynamics, determinants, and prognostic significance of zonulin in patients undergoing allo-HSCT. As demonstrated in the following sections, although we identified persistently elevated zonulin levels, zonulin did not emerge as a significant predictor of allo-HSCT–related outcomes.

## 2. Results

### 2.1. Demographic and Clinical Characteristics

The number of patients meeting the inclusion criteria (eligible patients included individuals undergoing any type of allo-HSCT except umbilical cord blood transplantation) was 140. These patients had 477 blood samples collected throughout predetermined allo-HSCT time points (D−7 [7 days prior to allo-HSCT], D0, D+30, D+60, D+90, D+180, and at the time of GvHD suspicion). Overall, most samples (70.2%; *n* = 335) were collected during the early time points (D−7 to D+30; see Supplementary Table S1 for details on the number of samples per time point and center). The demographics and clinical characteristics are shown in Table 1. Overall, the median age was 41 years (range, 12–73), and most patients were male (*n* = 79, 56.4%). The most common underlying diagnosis was leukemia (*n* = 100, 73.5%). In our overall cohort, mean follow-up time was 11 months (SD [Standard Deviation] ± 9 months).

### 2.2. Zonulin Dynamics During Allo-HSCT

**Figure 1:**
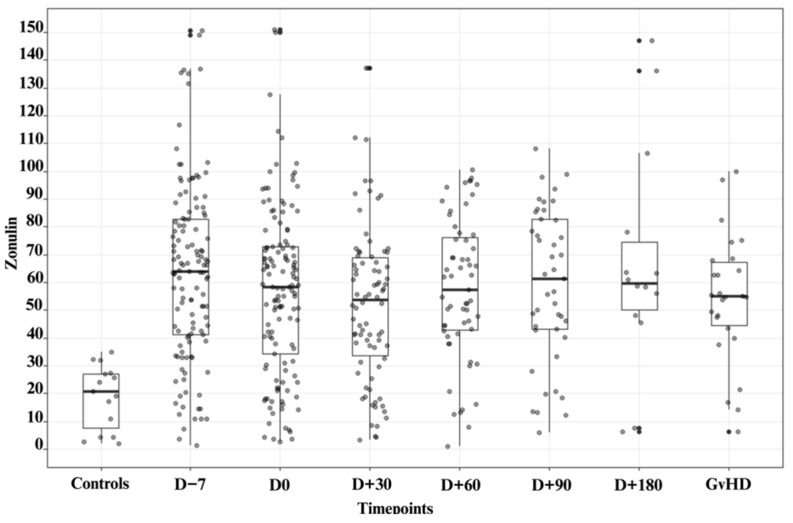
Temporal dynamics of zonulin during the allo-HSCT course (analyzed using paired Wilcoxon tests). D = day; GvHD = graft-versus-host disease.

Zonulin dynamics during allo-HSCT is shown in Figure 1. We also included a control group of 15 healthy adults, defined as individuals without chronic inflammatory diseases or recent use of antibiotics, anti-inflammatory drugs, or immunosuppressants, to better characterize zonulin dynamics. Overall, patients showed no significant difference in zonulin levels over the allo-HSCT course. However, when compared with the healthy control group (21 ng/mL), zonulin levels were persistently elevated at all allo-HSCT time points: D−7 (64 ng/mL; *p* < 0.0001), D0 (58 ng/mL, *p* < 0.0001), D+30 (54 ng/mL; *p* = 0.0003), D+60 (57 ng/mL; *p* < 0.0001), D+90 (61 ng/mL; *p* < 0.0001), D+180 (60 ng/mL; *p* = 0.0024), and GvHD (55 ng/mL; *p* = 0.0001). We also assessed zonulin dynamics over the allo-HSCT course stratified by the presence of GvHD and GI (Gastrointestinal)-GvHD (see Supplementary Figure S1A,B). In both analyses, no significant differences in zonulin trajectories were identified.

### 2.3. Predictors of Increased Zonulin Levels

To identify predictors of increased zonulin levels, patients were stratified at each time point into high- and low-zonulin groups based on the median zonulin value at the corresponding time point. Only early time points (up to D+30) were included in this analysis, given our interest in evaluating early zonulin levels as a potential prognostic biomarker. Among the variables analyzed as potential predictors of increased zonulin levels, at D−7, D0 and D+30, none were identified as significant (see Table 2). Additionally, none of the variables were significant predictors when analyzing Δ(delta)Zonulin (the difference between D0 and D−7).

### 2.4. Associations Between Zonulin and Clinical Outcomes

Among the 140 patients included in this study, 31 (23.0%) died during the follow-up period, 60 (45.0%) were diagnosed with GvHD, including 24 (17.0%) with GI GvHD, and 54 (38.6%) had BSI (bloodstream infection). Associations between each outcome and zonulin levels were evaluated by analyzing zonulin both as a continuous variable and as a categorical variable (low vs. high). For the categorical analyses, patients were stratified according to the median zonulin value at each time point. In addition, when zonulin was analyzed as a categorical variable, sensitivity, specificity, and predictive values were calculated for each outcome (see Supplementary Table S2).

#### 2.4.1. Associations Between Zonulin and GvHD

**Figure 2:**
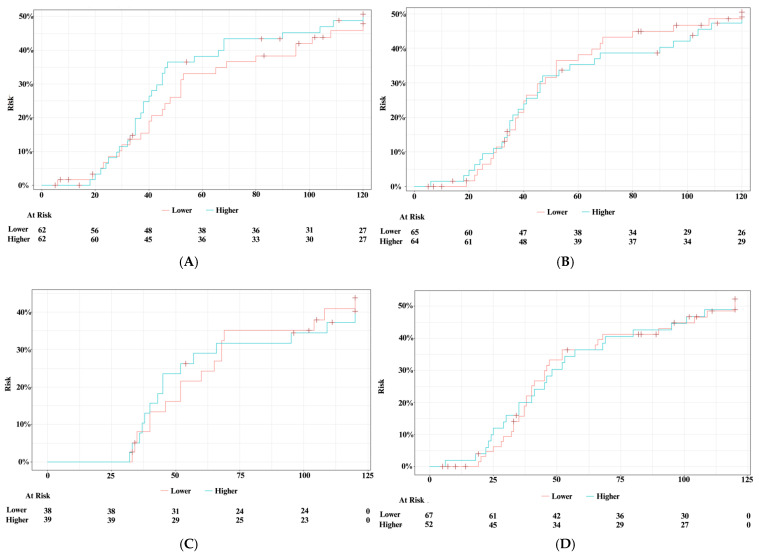
(**A**) Association between zonulin levels (low [red] vs. high [blue]) and the cumulative incidence of GvHD at D−7 (analyzed using Cox proportional hazards regression). D = day; GvHD = graft-versus-host disease. (**B**) Association between zonulin levels (low [red] vs. high [blue]) and the cumulative incidence of GvHD at D0 (analyzed using Cox proportional hazards regression). D = day; GvHD = graft-versus-host disease. (**C**) Association between zonulin levels (low [red] vs. high [blue]) and the cumulative incidence of GvHD at D+30 (analyzed using Cox proportional hazards regression). D = day; GvHD = graft-versus-host disease. (**D**). Association between zonulin levels (low [red] vs. high [blue]) and the cumulative incidence of GvHD using ΔZonulin (D0 minus D−7. D = day; analyzed using Cox proportional hazards regression). D = day; GvHD = graft-versus-host disease.

**Figure 3:**
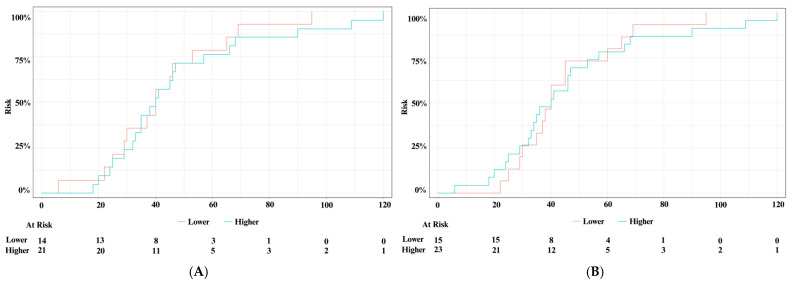

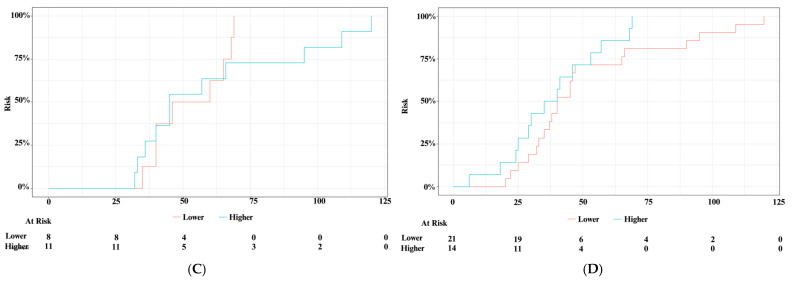
(**A**). Association between zonulin levels (low [red] vs. high [blue]) and the cumulative incidence of severe GvHD at D−7 (analyzed using Cox proportional hazards regression). D = day; GvHD = graft-versus-host disease. (**B**) Association between zonulin levels (low [red] vs. high [blue]) and the cumulative incidence of severe GvHD at D0 (analyzed using Cox proportional hazards regression). D = day; GvHD = graft-versus-host disease. (**C**) Association between zonulin levels (low [red] vs. high [blue]) and the cumulative incidence of severe GvHD at D+30 (analyzed using Cox proportional hazards regression). D = day; GvHD = graft-versus-host disease. (**D**) Association between zonulin levels (low [red] vs. high [blue]) and the cumulative incidence of severe GvHD using ΔZonulin (D0 minus D−7; (analyzed using Cox proportional hazards regression). D = day; GvHD = graft-versus-host disease.

We first categorized patients based on the median zonulin level into high vs. low zonulin groups. No significant associations were identified with the cumulative incidence of GvHD (D−7: HR [Hazard Ratio] 1.13; 95% CI [Confidence Interval] 0.67–1.91; *p* = 0.6; D0: HR 0.96; 95% CI 0.58–1.59; *p* = 0.9; D+30: HR 0.95; 95% CI 0.47–1.91; *p* > 0.9; ΔZonulin: HR 0.95; 95% CI 0.56–1.60; *p* = 0.8; see Figure 2A–D), and severe GvHD (D−7: HR 0.74; 95% CI 0.37–1.47; *p* = 0.4; D0: HR 0.88; 95% CI 0.45–1.71; *p* = 0.7; D+30: HR 0.75; 95% CI 0.28–1.00; *p* = 0.6; ΔZonulin: HR 1.54; 95% CI 0.76–3.14; *p* = 0.2 see Figure 3A–D). Similarly, no significant associations with the cumulative incidence of GvHD or severe GvHD were found when zonulin levels were analyzed as a continuous variable (see Table 3).

#### 2.4.2. Associations Between Zonulin and Overall Survival

**Figure 4:**
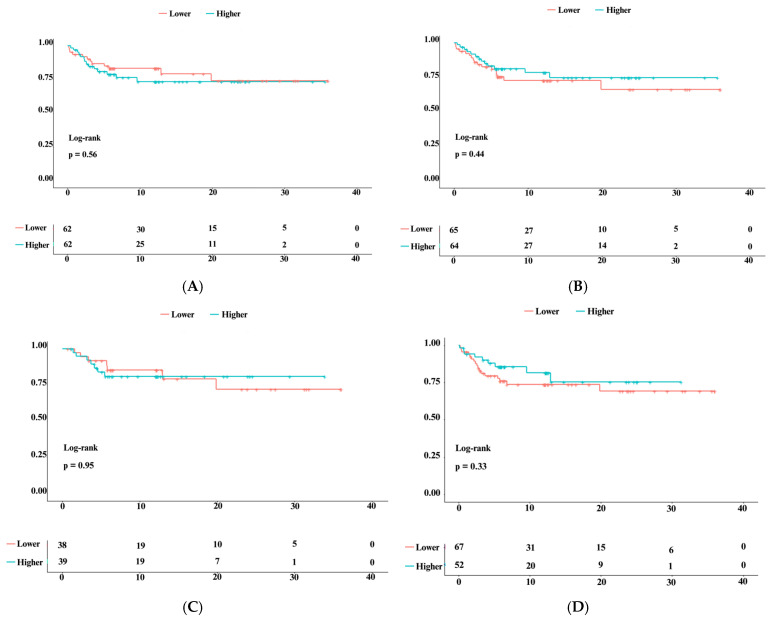
(**A**) Association between zonulin levels (low [red] vs. high [blue]) and overall survival at D−7 (analyzed using log-rank and Kaplan–Meier). D = day. (**B**) Association between zonulin levels (low [red] vs. high [blue]) and overall survival at D0 (analyzed using log-rank and Kaplan–Meier). D = day. (**C**) Association between zonulin levels (low [red] vs. high [blue]) and overall survival at D+30 (analyzed using log-rank and Kaplan–Meier). D = day. (**D**). Association between zonulin levels (low [red] vs. high [blue]) and overall survival using ΔZonulin (D0 minus D−7; (analyzed using log-rank and Kaplan–Meier). D = day.

Zonulin was not significantly associated with overall survival when analyzed as a categorical variable based on the median value (D−7: HR 1.26; 95% CI 0.58–2.72; *p* = 0.6; D0: HR 0.75; 95% CI 0.37–1.55; *p* = 0.4; D+30: HR 1.03; 95% CI 0.36–2.96; *p* > 0.9; ΔZonulin: HR 0.67; 95% CI 0.30–1.51; *p* = 0.3; see Figure 4). When analyzed as a continuous variable (see Table 3), zonulin levels were not significantly associated with overall survival at the evaluated time points.

#### 2.4.3. Associations Between Zonulin and BSI

Similarly to the other clinical endpoints, there was no significant association between zonulin levels and BSI when patients were stratified as high versus low at any of the time points (D−7: OR 0.77; 95% CI 0.37–1.57; *p* = 0.5; D0: OR 1.09; 95% CI 0.54–2.21; *p* = 0.8; D+30: OR 1.64; 95% CI 0.25–13.4; *p* = 0.6; ΔZonulin: HR 0.61; 95% CI 0.29–1.29; *p* = 0.2). Zonulin was also not significantly associated with BSI when analyzed as a continuous variable (see Table 3).

## 3. Discussion

In this multicenter, prospective study evaluating the dynamics, predictors, and prognostic significance of zonulin in the allo-HSCT setting, we identified several major findings. First, zonulin levels remained consistently elevated throughout the allo-HSCT course. Second, no clinical or procedure-related characteristics appeared to be good predictors of increased zonulin levels. Finally, although zonulin levels were persistently elevated, they did not appear to be a useful prognostic marker.

An interesting finding of this study is that zonulin levels remained consistently elevated throughout the entire course of allo-HSCT, suggesting persistently gastrointestinal barrier damage. This is partially supported by prior studies [1,2,3,11,14,18,19,20,21,22,23]. Although intestinal permeability has been consistently reported to be increased in patients undergoing allo-HSCT, several studies have shown significant temporal dynamics [2,11,13,14,18,22,24,25,26]. For instance, in a study including 80 allo-HSCT patients, the lactulose:rhamnose (L:R) ratio was used to assess intestinal permeability at the following time points: baseline (preconditioning), D+7, and D+30 [18]. Compared with baseline, the L:R ratio significantly increased at D+7 (0.19 vs. 0.86; *p* < 0.0001) and at D+30 (0.19 vs. 0.40; *p* = 0.014) [18]. However, there was a significant decrease at D+30 compared with D+7 (0.86 vs. 0.40; *p* = 0.014) [18]. Similar findings were reported in other studies using the L:R ratio to evaluate intestinal permeability [13,24,25]. In contrast, conflicting findings in intestinal permeability dynamics were reported when biomarkers such as occludin and calprotectin were used as surrogate markers [1,11,18]. In a study including 31 allo-HSCT patients, calprotectin was measured at the following time points: D−7, D+7, D+14, and D+21 [1]. Calprotectin levels were significantly decreased compared with reference values, and no significant differences were observed among samples collected at different time points during the allo-HSCT course (D−7 vs. D+7: *p* = 0.374; D−7 vs. D+14; *p* value not reported; D−7 vs. D+21; *p* = 0.451) [1]. However, in a pediatric study, significant changes in calprotectin levels were observed during the allo-HSCT (Baseline (before allo-HSCT): 52.53 vs. D+90: 123 vs. D+180 90.05 ug/g; *p* < 0.001) [11]. Together with our findings, these studies highlight the challenges of assessing intestinal permeability during allo-HSCT.

These challenges are further compounded by evidence that distinct mechanisms may transiently contribute to increased intestinal permeability at different stages of the allo-HSCT course [21]. It is important to highlight that intestinal permeability is a complex concept that encompasses multiple gastrointestinal components, and each available methodology assesses only specific aspects of the intestinal barrier. In this context, the persistently elevated zonulin levels observed in our cohort may suggest sustained activation of the zonulin-mediated pathway throughout the allo-HSCT course, but not necessarily its direct relevance to the mechanisms underlying GvHD-related intestinal permeability. Alternatively, our findings may indicate that the zonulin pathway is already maximally activated prior to transplantation, thereby limiting its ability to provide incremental prognostic information. In contrast, a prior pediatric study including 100 allo-HSCT patients reported significant temporal changes in zonulin levels between pre-transplant and 3 months post-transplant (56.11 ± 15.43 vs. 90.69 ± 20.32; *p* = 0.001), as well as an association with GvHD severity (*p* = 0.002) [11]. Although these findings are not supported by our data, several important differences between the studies may account for these discrepancies. First, the pediatric study included only patients with complete longitudinal sampling at 3 and 6 months, which may have introduced a selection bias toward patients with fewer complications who survived longer after transplantation [11]. Second, patients with liver or renal dysfunction were excluded, potentially removing individuals with more severe allo-HSCT-related complications [11]. Third, the study population consisted exclusively of pediatric patients, whereas our cohort was predominantly adult [11]. Fourth, the authors did not provide detailed information regarding the zonulin assay used, and prior studies have demonstrated variability across different commercial kits [27,28,29]. Finally, there were differences in underlying diagnoses, with our cohort being largely composed of patients with malignant diseases, while the pediatric study primarily included non-malignant conditions such as hemophilia, immune disorders, and bone marrow failure syndromes [11]. Therefore, future studies should evaluate intestinal permeability during allo-HSCT using multiple complementary and validated methodologies. These complementary methodologies should aim to capture the distinct components of the intestinal barrier, including enterocyte mass (e.g., citrulline), enterocyte turnover (e.g., I-FABP [Intestinal Fatty Acid-Binding Protein]), paracellular permeability (e.g., zonulin, claudin, occludin, and L:R ratio), and transcellular transport (e.g., R:O [rhamnose:3-O-methyl-D-glucose] and X:O [xylose: 3-O-methyl-D-glucose] ratios). Only through such an approach will it be possible to accurately characterize the key pathways and mechanisms involved in intestinal barrier dysfunction in the allo-HSCT setting.

Another important finding of this study is that no patient- or procedure-related characteristics were identified as predictors of increased zonulin levels. Although serum zonulin has not been evaluated in prior adult allo-HSCT studies, predictors of increased intestinal permeability consistently reported in the literature include: (1) conditioning regimen [2,14,15,30], (2) dietary interventions [13,19,23], (3) intestinal microbiota [18,19], and (4) immune response [20,30,31]. Regarding the conditioning regimen, studies have shown that myeloablative conditioning, compared with other regimens, is associated with increased levels of 51Cr-EDTA (Chromium-51 labeled ethylenediaminetetraacetic acid) [2,14], and decreased levels of citrulline [30], and I-FABP [15]. Myeloablative conditioning, however, was not associated with changes in the L:R and L:M ratios [1,18]. Similarly, conflicting findings have been reported regarding the intestinal microbiota [18,19]. While both microbiota diversity indices and microbiota composition have been associated with the L:R ratio [18], no significant associations were observed when analyzing I-FABP, LBP (Lipopolysaccharide-binding protein), and sCD14 (Soluble cluster of differentiation 14) [19]. Taken together, these findings further highlight the complexity of assessing intestinal permeability in the allo-HSCT setting. Because each methodology evaluates specific components of the gastrointestinal barrier, the identified predictors may differ across approaches. Therefore, future studies should not only apply multiple complementary methodologies but also comprehensively assess potential predictive factors. Identifying predictors of increased intestinal permeability is clinically relevant, as these factors may represent therapeutic targets for interventions aimed at modulating intestinal barrier function.

The final major finding of this study is that zonulin levels were not significantly associated with overall survival, aGvHD, or BSI. The consistent observation of hazard ratios close to 1 with relatively narrow 95% confidence intervals, combined with our relatively large cohort (*n* = 140), supports the interpretation that this represents a true negative finding rather than the result of insufficient statistical power. This is partially supported by prior studies, which have reported conflicting results regarding the prognostic significance of intestinal permeability markers [1,2,3,11,15,19,22,23,24,25,31,32,33,34]. For instance, while intestinal permeability markers were not associated with overall survival [18,19,32,34], significant associations have been reported when analyzing aGvHD [1,2,3,23]. Although hypotheses exist regarding translocation of the intestinal microbiota as a source of BSI in the allo-HSCT setting, studies have consistently reported non-significant associations with several distinct permeability markers, including the L:R ratio, L:M ratio, calprotectin, beta-defensin-2, haptoglobin genotype, and LBP [1,3,18,31,34]. Interestingly, in a study evaluating the prognostic significance of serum zonulin in pediatric patients undergoing allo-HSCT, increased zonulin levels were associated with GvHD severity (110.05 vs. 62.33 ng/mL; *p* = 0.002), diarrhea (100.23 vs. 72.22 ng/mL; *p* = 0.036), and mucositis (105.81 vs. 66.22 ng/mL; *p* = 0.029) [11]. Overall, our findings, together with prior studies, suggest that the prognostic significance of intestinal permeability during the allo-HSCT course remains inconclusive. Several methodological limitations, including small sample sizes and heterogeneity in permeability assessment methods, may contribute to these inconsistent results. Therefore, future studies should evaluate intestinal permeability in larger cohorts using standardized methodologies.

The primary strength of this study is the longitudinal assessment of zonulin levels in a large cohort of patients undergoing allo-HSCT, particularly when compared with prior studies of intestinal permeability in this setting [1,2,14,18,20,21,22,35]. Additionally, to our knowledge, this is the first study to systematically evaluate serum zonulin dynamics and prognostic significance in the adult allo-HSCT setting. This study, however, has some limitations. First, prior studies have demonstrated concerns regarding the sensitivity and specificity of commercially available zonulin kits [27,28,29]. Evidence suggests that these assays may not specifically measure zonulin (prehaptoglobin-2) or correlate with haptoglobin genotypes, but instead detect a group of structurally related proteins, including complement components such as C3 and properdin [27,28,29]. However, the zonulin ELISA kit used in our study reports no significant cross-reactivity with structurally related proteins according to the manufacturer’s specifications. Nonetheless, independent validation studies assessing its analytical specificity are still needed. Second, although predefined time points for sample collection were established, there was a significant decrease in the number of blood samples collected after D+30. This reduction may be attributable to several factors, including withdrawal from follow-up and mortality. This reduction may introduce attrition bias and substantially decrease the number of available samples at later timepoints, thereby limiting our ability to detect significant longitudinal changes in zonulin levels and to assess the prognostic significance of late timepoint samples for allo-HSCT outcomes. Finally, we did not validate zonulin levels using the gold-standard method for assessing intestinal permeability (namely Ussing chamber assays and/or probe-based methodologies). Notwithstanding these limitations, this study provides novel insights into the potential role of zonulin in the allo-HSCT setting. Future studies should simultaneously evaluate multiple complementary measures of intestinal permeability to comprehensively assess the different components of the intestinal barrier. Additionally, further studies are needed to validate zonulin assays in the context of allo-HSCT. Only with such validation studies can accurate conclusions be drawn regarding the prognostic significance of zonulin in the allo-HSCT setting.

## 4. Materials and Methods

### 4.1. Study Design and Ethical Aspects

This was a prospective, observational, multicenter cohort study of patients under-going allo-HSCT. The study was approved on 1 December 2021, by the Research Ethics Committee of São Paulo State University (protocol number 5.138.190/2021) and was conducted in accordance with the Declaration of Helsinki. Adult participants provided written informed consent at the beginning of the study. For pediatric patients, both the patient and their legal guardian agreed to participate and provided signed informed consent forms. The inclusion criteria were patients aged ≥12 years undergoing allo-HSCT who provided at least one blood sample during the 6-month follow-up period. Transplants from related donors (including matched related donors and haploidentical donors) as well as unrelated donors (including matched and mismatched unrelated donors) were included. The exclusion criteria were: (1) patients undergoing autologous HSCT or umbilical cord blood transplantation, and (2) patients without adequate clinical follow-up or biological samples. Fifteen healthy adult controls without chronic inflammatory diseases or recent use of antibiotics, anti-inflammatory drugs, or immunosuppressants also signed informed consent forms and agreed to participate in the study.

### 4.2. Sample Collection and Zonulin Measurement

Blood samples (5 mL) were collected from participants into K2-EDTA tubes at four different Brazilian hospitals (Hospital de Base of the Fundação Faculdade Regional de Medicina [HB-FUNFARME], Hospital Amaral Carvalho [HAC], Hospital de Câncer de Barretos [HCB], and Hospital Beneficência Portuguesa de São Paulo [BP]). Plasma was separated by centrifugation at 1372× *g* for 10 min and temporarily stored at −20 °C until transport to the central laboratory, where it was stored at −80 °C until analysis. Plasma zonulin concentrations were measured using a commercial enzyme-linked immuno-sorbent assay (Human Zonulin ELISA Kit, Elabscience, Houston, TX, USA), according to the manufacturer’s instructions. Although limitations in zonulin specificity have been described for some commercial ELISA assays, the manufacturer of the ELISA used in our study reports no significant cross-reactivity with structurally related proteins [27,28]. Optical density (OD) was read at 450 nm using a spectrophotometer. Calibration curves were constructed in Excel using the equation y = ax + b, in which x and y represented OD and concentration, respectively. Zonulin concentrations were calculated by converting OD values into ng/mL. Samples were collected longitudinally at seven time points: prior to the conditioning regimen (D−7), on the day of stem cell infusion (D0), 30 days after transplantation (D+30), D+60, D+90, D+180, and at the time of acute graft-versus-host disease (aGvHD) diagnosis. Although efforts were made to collect samples at these exact time points, some variability occurred due to rescheduling of follow-up visits and intercurrent clinical events.

### 4.3. Clinical Data and Outcomes

Study data were collected and managed using REDCap (Research Electronic Data Capture) electronic data capture tools hosted at HCB [36,37]. REDCap is a secure, web-based software platform designed to support data capture for research studies, providing (1) an intuitive interface for validated data capture; (2) audit trails for tracking data manipulation and export procedures; (3) automated export procedures for seamless data downloads to common statistical packages; and (4) procedures for data integration and interoperability with external sources [36,37]. Variables collected included detailed demographic characteristics and transplant-related data. Outcomes of interest were overall survival, aGvHD (cumulative incidence), and BSI. Acute GvHD was diagnosed clinically, confirmed histopathologically by biopsy whenever possible, and graded according to the MAGIC criteria [38]. As in previous studies, the onset of aGvHD was defined based on the appearance of clinical symptoms (clinical suspicion), as determined by transplant specialists based on manifestations commonly associated with acute GvHD, including skin rash, nausea, vomiting, diarrhea, and jaundice [39,40]. BSI was defined by the presence of positive blood cultures within the first 90 days after allo-HSCT.

### 4.4. Statistical Analysis

We initially generated descriptive statistics stratified by center. To evaluate the longitudinal dynamics of zonulin levels during allo-HSCT, paired Wilcoxon tests were used. To identify predictors of increased zonulin levels, logistic regression analysis was performed. Based on prior literature, the following potential predictors were included: (1) demographic characteristics (participating center, age, weight, height, and sex); (2) history of previous HSCT; (3) use of total body irradiation (TBI) in the conditioning regimen; (4) type of conditioning (myeloablative, non-myeloablative, or reduced-intensity); (5) underlying disease (leukemias versus other indications); and (6) use of antibiotics [1,18,19]. Antibiotic data was available in two centers (HB-FUNFARME and BP).

To evaluate the association between zonulin levels and clinical outcomes, Kaplan–Meier analysis and the log-rank test were used for overall survival, and Cox proportional hazards regression was used to assess the cumulative incidence of aGvHD. For GvHD, given our interest in evaluating zonulin as a predictive biomarker, we considered only events occurring after sample collection. Accordingly, for analyses of samples collected at D+30, we excluded patients who developed GvHD prior to that time point. To assess the relationship between zonulin levels and BSI, we used logistic regression models. For all the outcomes, these associations were analyzed using unadjusted models and considering zonulin both as a continuous and as a categorical variable. When analyzed as a categorical variable, zonulin levels were dichotomized based on the median value at each time point, classifying patients into high and low groups. Additionally, given our interest in evaluating zonulin as a prognostic biomarker, we also assessed predictive performance (sensitivity, specificity, and predictive values) when zonulin was analyzed as a categorical variable. Given our goal of evaluating zonulin as a predictive biomarker, we assessed only zonulin levels at D−7, D0, and D+30. We also evaluated ΔZonulin (the difference between D0 and D−7). All statistical analyses were performed using R software (version 4.5.3), and *p* values < 0.05 were considered statistically significant.

## Figures and Tables

**Table 1:** Demographic and Clinical Characteristics.

Variables	HB-FUNFARME (N = 40)	HAC (N = 48)	HCB (N = 10)	BP (N = 42)	Total (140)
**Age (years)**					
Median (Range)	39 (18–66)	43 (13–68)	43 (19–61)	43 (12–73)	41 (12–73)
Mean (SD)	41 (14)	41 (15)	41 (15)	46 (18)	42 (16)
**Biological Sex**					
Female	14 (35.0%)	22 (45.8%)	6 (60.0%)	19 (45.2%)	61 (43.6%)
Male	26 (65.0%)	26 (54.2%)	4 (40.0%)	23 (54.8%)	79 (56.4%)
**Ethnicity**					
Afro-descendants	9 (22.5%)	25 (52.1%)	4 (40.0%)	14 (33.3%)	52 (37.1%)
Asian	1 (2.5%)	0 (0.0%)	0 (0.0%)	1 (2.4%)	2 (1.4%)
Caucasian	30 (75.0%)	23 (47.9%)	6 (60.0%)	27 (64.3%)	86 (61.4%)
**Weight (kg)**					
Median (Range)	79 (51–115)	73 (47–109)	68 (43–81)	73 (46–130)	73 (43–130)
Mean (SD)	78 (15)	76 (15)	66 (13)	74 (19)	75 (16)
**BMI**					
Median (Range)	26.1 (15.8–40.7)	26.5 (17.5–37.6)	22.8 (17.5–38.0)	25.2 (17.0–38.4)	25.8 (15.8–40.7)
Mean (SD)	26.6 (4.9)	27.1 (4.6)	24.7 (6.2)	26.3 (5.1)	26.5 (5.0)
**Height (cm)**					
Median (Range)	173 (151–191)	167 (144–195)	166 (146–188)	166 (150–189)	168 (144–195)
Mean (SD)	171 (10)	167 (10)	165 (12)	168 (10)	168 (10)
**Underlying Diagnosis**					
Leukemias	30 (75.0%)	36 (75.0%)	6 (60.0%)	31 (73.8%)	103 (73.6%)
Lymphomas	2 (5.0%)	1 (2.1%)	3 (30.0%)	1 (2.4%)	7 (5.0%)
Others	8 (20.0%)	11 (22.9%)	1 (10.0%)	10 (23.8%)	30 (21.4%)
**TBI**					
No-TBI	29 (72.5%)	21 (43.8%)	2 (20.0%)	18 (42.9%)	70 (50.0%)
Yes-TBI	11 (27.5%)	27 (56.3%)	8 (80.0%)	24 (57.1%)	70 (50.0%)
**Conditioning Regimen**					
Myeloablative	14 (35.0%)	23 (47.9%)	2 (20.0%)	12 (28.6%)	51 (36.4%)
Non Myeloablative	5 (12.5%)	2 (4.2%)	0 (0.0%)	14 (35.9%)	21 (15.0%)
Reduced Intensity	21 (52.5%)	23 (47.9%)	8 (80.0%)	16 (38.1%)	68 (48.6%)
**HLA Match Status**					
MRD	18 (45.0%)	13 (27.1%)	1 (10.0%)	10 (23.8%)	42 (30.0%)
MUD	1 (2.5%)	9 (18.8%)	1 (10.0%)	6 (14.3%)	17 (12.1%)
Haploidentical	21 (52.5%)	23 (47.9%)	6 (60.0%)	21 (50.0%)	71 (50.7%)
MMUD	0 (0.0%)	3 (6.3%)	0 (0.0%)	2 (4.8%)	5 (3.6%)
Mismatched Related Donor (5–10 HLA match)	0 (0.0%)	0 (0.0%)	2 (20.0%)	3 (7.1%)	5 (3.6%)
**Donor Sex**					
Male	24 (60.0%)	34 (70.8%)	7 (70.0%)	25 (59.5%)	90 (64.3%)
Female	16 (40.0%)	14 (29.2%)	3 (30.0%)	17 (40.5%)	50 (35.7%)
**Stem Cell Source**					
Peripheral Blood	37 (92.5%)	28 (58.3%)	9 (90.0%)	36 (85.7%)	110 (78.6%)
Bone Marrow	3 (7.5%)	20 (41.7%)	1 (10.0%)	5 (11.9%)	29 (20.7%)
Others	0 (0.0%)	0 (0.0%)	0 (0.0%)	1 (2.4%)	1 (0.7%)
**Prior Stem Cell Transplant**					
No	39 (97.5%)	45 (93.8%)	6 (60.0%)	40 (95.2%)	130 (92.9%)
Yes	1 (2.5%)	3 (6.3%)	4 (40.0%)	2 (4.8%)	10 (7.1%)

BMI = Body Mass Index; HLA = Human Leukocyte Antigens; MMUD = Mismatched Unrelated Donor; MRD = Matched Related Donor; MUD = Matched Unrelated Donor; N = Number; SD = Standard Deviation; TBI = Total Body Irradiation; HB-FUNFARME = Hospital de Base of the Fundação Faculdade Regional de Medicina; HAC = Hospital Amaral Carvalho; HCB = Hospital de Câncer de Barretos; BP = Hospital Beneficência Portuguesa de São Paulo.

**Table 2:** Predictors of Increased Zonulin Levels at Different Time Points in the Overall Cohort of Allo-HSCT Patients.

	D−7		D0		D+30		∆P	
Variables	OR (95% CI) *	*p* Value	OR (95% CI) *	*p* Value	OR (95% CI) *	*p* Value	OR (95% CI) *	*p* Value
**Hospital**								
HAC	–		–		–		–	
BP	0.64 (0.27, 1.51)	0.3	0.61 (0.26, 1.40)	0.2	0.66 (0.20, 2.10)	0.5	2.11 (0.88, 5.14)	0.10
HB-FUNFARME	1.67 (0.65, 4.46)	0.3	1.06 (0.42, 2.69)	>0.9	1.56 (0.50, 4.96)	0.4	1.57 (0.58, 4.26)	0.4
HCB	0.22 (0.03, 0.99)	0.072	0.35 (0.07, 1.41)	0.2	2.69 (0.49, 21.2)	0.3	0.43 (0.06, 1.95)	0.3
**Weight (Kg)**	0.99 (0.97, 1.01)	0.4	1.01 (0.99, 1.04)	0.2	1.01 (0.99, 1.04)	0.3	1.02 (1.00, 1.04)	0.11
**Height (Cm)**	0.97 (0.94, 1.01)	0.14	0.99 (0.96, 1.02)	0.5	0.98 (0.98, 1.02)	0.4	1.01 (0.98, 1.05)	0.6
**Biological Sex**								
Female	–		–		–		–	
Male	0.59 (0.29, 1.21)	0.2	0.59 (0.29, 1.18)	0.14	0.44 (0.17, 1.10)	0.082	0.82 (0.40, 1.71)	0.6
**Prior Stem Cell Transplant**								
No	–		–		–		–	
Yes	1.00 (0.26, 3.78)	>0.9	1.58 (0.43, 6.44)	0.5	3.27 (0.70, 23.4)	0.2	0.85 (0.21, 3.13)	0.8
**TBI**								
No-TBI	–		–		–		–	
Yes-TBI	0.68 (0.33, 1.37)	0.3	0.63 (0.31, 1.25)	0.2	0.85 (0.34, 2.09)	0.7	0.69 (0.33, 1.44)	0.3
**Conditioning Regimen**								
Myeloablative	–		–		–		–	
Reduced Intensity	0.94 (0.43, 2.05)	0.9	0.64 (0.29, 1.36)	0.2	1.00 (0.38, 2.65)	>0.9	0.95 (0.42, 2.06)	0.9
Non Myeloablative	0.66 (0.22, 1.95)	0.5	0.69 (0.23, 2.03)	0.5	1.20 (0.30, 5.00)	0.9	1.12 (0.36, 3.49)	0.8
**Age at Allo-HSCT**	1.00 (0.98, 1.02)	>0.9	1.00 (0.97, 1.02)	0.7	1.00 (1.00, 1.00)	0.8	1.00 (0.98, 1.02)	>0.9
**Underlying Diagnosis**								
Leukemia	–		–		–			
Others	0.79 (0.36, 1.72)	0.5	1.11 (0.50, 2.46)	0.8	1.27 (0.46, 3.59)	0.7	0.59 (0.25, 1.34)	0.2
**Prior Antibiotics Use**	N/A	N/A	0.47 (0.14, 1.40)	0.2	0.70 (0.28, 1.70)	0.4	N/A	N/A
**Antibiotics Use for ≥3D Prior to Sample Collection**	N/A	N/A	1.25 (0.64, 2.55)	0.5	0.70 (0.28, 1.70)	0.4	N/A	N/A
Beta-Lactams	N/A	N/A	0.80 (0.19, 3.16)	0.7	0.63 (0.25, 1.54)	0.3	N/A	N/A
Fluoroquinolones	N/A	N/A	1.02 (0.04, 26.1)	>0.9	2.06 (0.38, 15.5)	0.4	N/A	N/A
Glycopeptides	N/A	N/A	0.50 (0.02, 5.35)	0.6	1.33 (0.44, 4.15)	0.6	N/A	N/A
Anti-anaerobics	N/A	N/A	0.80 (0.19, 3.16)	0.7	0.63 (0.25, 1.54)	0.3	N/A	N/A

Allo-HSCT = Allogeneic hematopoietic stem cell transplantation; CI = Confidence interval; D = day; N/A = Not applicable; OR = Odds ratio; TBI = Total Body Irradiation; ∆P = Difference between permeability in periods D−7 and D0; * = Logistic regression analysis was performed to identify predictors of increased zonulin levels.

**Table 3:** Associations Between Zonulin and Clinical Outcomes.

	D−7		D0		D+30	
	HR (95% CI)	*p* Value	HR (95% CI)	*p* Value	HR (95% CI)	*p* Value
GvHD	1.00 (1.00, 1.01) *	0.6	1.00 (1.00, 1.01) ^†^	0.4	1.00 (0.99, 1.01)	0.7 ^Î^
Severe GvHD	1.00 (0.98, 1.01) **	0.5	1.00 (0.99, 1.01) ^††^	0.6	0.99 (0.97, 1.00)	0.12 ^ÎÎ^
Overall Survival	1.00 (0.99, 1.01) *	>0.9	1.00 (0.99, 1.01) ^†^	>0.9	1.01 (0.99, 1.02)	0.5 ^Î^
BSI	1.00 (0.99, 1.01) *	>0.9	1.00 (0.99, 1.01) ^†^	0.8	1.00 (0.97, 1.03)	>0.9 ^ÎÎÎ^

BSI = Bloodstream infection; CI = Confidence interval; D = Day; GvHD = Graft-versus-graft disease; HR = Hazard Ratio; * = 124 patients included in this analysis; ** = 35 patients included in this analysis; ^†^ = 129 patients included in this analysis; ^††^ = 38 patients included in this analysis; ^Î^ = 77 patients included in this analysis; ^ÎÎ^ = 19 patients included in this analysis; ^ÎÎÎ^ = 82 patients included in this analysis.

## Data Availability

The data associated with this study will not be made publicly available. However, data associated with this project may be shared upon specific request in a deidentified manner and in accordance with appropriate data-sharing requirements.

## Supplementary Materials

The following supporting information can be downloaded at: https://www.mdpi.com/article/10.3390/ijms27114659/s1.

## Abbreviations

The following abbreviations are used in this manuscript:

aGvHD	Acute Graft-versus-Host Disease
allo-HSCT	Allogeneic Hematopoietic Stem Cell Transplantation
ATB	Antibiotic
BMI	Body Mass Index
BP	Hospital Beneficência Portuguesa de São Paulo
BSI	Bloodstream Infection
CI	Confidence Interval
D	Day
ELISA	Enzyme-Linked Immunosorbent Assay
GI-GvHD	Gastrointestinal Graft-versus-Host Disease
GvHD	Graft-versus-Host Disease
HAC	Hospital Amaral Carvalho
HB-FUNFARME	Hospital de Base da Fundação Faculdade Regional de Medicina
HCB	Hospital de Câncer de Barretos
HLA	Human Leukocyte Antigens
HR	Hazard Ratio
HSCT	Hematopoietic Stem Cell Transplantation
I-FABP	Intestinal Fatty Acid-Binding Protein
K2-EDTA	Dipotassium Ethylenediaminetetraacetic Acid
LBP	Lipopolysaccharide-Binding Protein
L:M ratio	Lactulose:Mannitol Ratio
L:R ratio	Lactulose:Rhamnose Ratio
MAGIC	Mount Sinai Acute GVHD International Consortium
MMUD	Mismatched Unrelated Donor
MRD	Matched Related Donor
MUD	Matched Unrelated Donor
N	Number
N/A	Not Applicable
NR	Not Reported
OD	Optical Density
OR	Odds Ratio
REDCap	Research Electronic Data Capture
sCD14	Soluble CD14
SD	Standard Deviation
TBI	Total Body Irradiation
