# Supplementary material for: Unraveling the Role of Zonulin in Allogeneic Hematopoietic Stem Cell Transplantation: A Multicenter Study
Source: Int J Mol Sci. 2026 May 22;27(11):4659. doi: 10.3390/ijms27114659 (PMC13256533; doi:10.3390/ijms27114659)
Supplement: Supplementary file 1 [file ijms-27-04659-s001.zip › ijms-4287266-supplementary/ijms-4287266-supplementary.pdf]

Supplementary Tables

Supplementary Table S1 – Number of samples per time point								
**Center**	**D-7**	**D0**	**D30**	**D60**	**D90**	**D180**	**aGvHD**	**Total**
HCB	10	10	8	6	6	2	2	44
HAC	47	47	30	15	11	4	0	154
HB-FUNFARME	29	30	23	16	10	8	7	123
BP	38	42	21	22	16	0	17	156
Total	124	129	82	59	43	14	26	477

aGvHD: acute graft-versus-host-disease; BP = Hospital Beneficência Portuguesa de São Paulo; D = day; HCB = Hospital de Câncer de Barretos; HAC = Hospital Amaral Carvalho; HB-FUNFARME = Hospital de Base of the Fundação Faculdade Regional de Medicina.

Supplementary Table S2 – Diagnostic Performance of Zonulin for Allo-HSCT–Related Outcomes												
	GvHD			Severe GvHD			Overall Survival			BSI		
	D−7	D0	D+30	D−7	D0	D+30	D−7	D0	D+30	D−7	D0	D+30
Sensitivity	0.526	0.500	0.484	0.600	0.605	0.579	0.538	0.433	0.500	0.462	0.509	0.600
Specificity	0.522	0.507	0.478	0.539	0.549	0.517	0.510	0.485	0.492	0.472	0.513	0.523
Positive Predictive Value	0.484	0.469	0.385	0.339	0.359	0.282	0.226	0.203	0.179	0.387	0.422	0.125
Negative Predictive Value	0.565	0.538	0.579	0.774	0.769	0.789	0.806	0.738	0.816	0.548	0.600	0.920

Allo-HSCT = Allogeneic hematopoietic stem cell transplantation; BSI = Bloodstream infection; GvHD = Graft-versus-host disease

Supplementary Figures

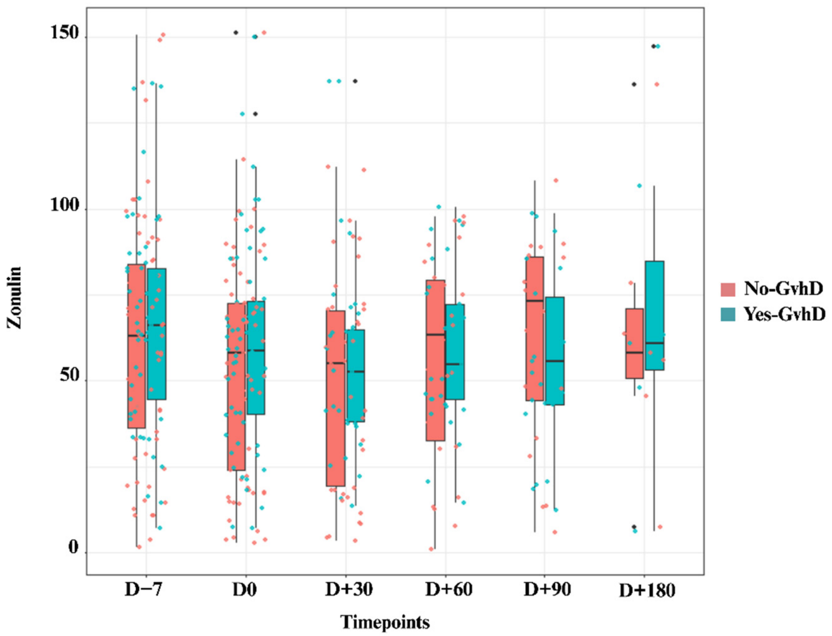

Supplementary Figure S1. (A)

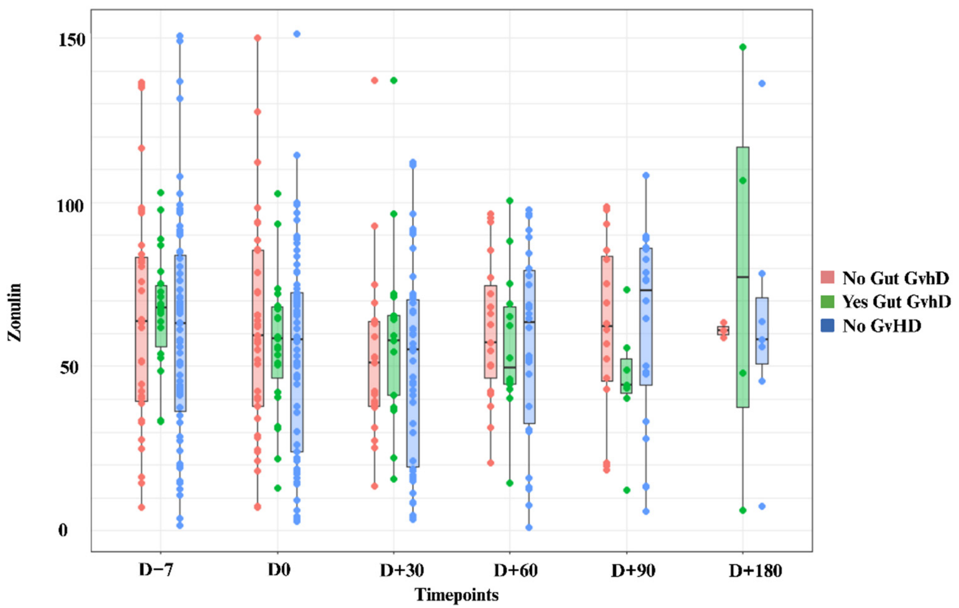

Supplementary Figure S1. (B)

Supplementary Figure S1. (A) Temporal dynamics of zonulin throughout the allo-HSCT course stratified according to the presence of GvHD. **(B)** Temporal dynamics of zonulin throughout the allo-HSCT course stratified according to the presence of gut GvHD. D = day; GvHD = graft-versus-host disease.
